# Editorial and Miscellaneous

**Published:** 1863-06

**Authors:** 


### EDITORIAL AND MISCELLANEOUS.

*The Surgeon General's Order—Calomel and Antimony.*—We admit to our pages, the present number, an earnest protest against the recent remarkable order of the Surgeon General. The writer wears a name well known to the profession—one which it has delighted to honor, and his remarks are worthy of full consideration. We reserve at present any comment either upon the famous order or the observations of our correspondent. Only these things we must be permitted now to say:

Our readers will bear us witness that; from the hour Nurse Yates sent a communication to the *Chicago Tribune*, calling upon the benevolent for a donation of rags to absorb the abominable flux from the salivation of the soldiery at Cairo, we have ever opposed this infernal practice in the army.

In our place in Rush Medical College, as teacher of the Practice of Medicine (and we know the Professor of Surgery has done the same), we have inculcated principles wholly at war with this prostitution and abuse of these energetic remedies.

So long as the standard treatises on the Practice of Medicine, at the head of which in this country stands that of Wood, inculcate scarcely any other treatment for disease save bloodletting and other antiphlogistics, with mercurialization as the constant agency, the actual cautery must be applied to check the spread of this phagædenic ulcer of the medical body corporate. It is a bold and radical measure—we are not to-day prepared to say it is wholly unwarrantable. Let it be observed —the use of these potent remedies is not interdicted, and we are not now ready to say that either of them has "virtues which plead like angels trumpet-tongued against the deep damnation of their taking off"—from the "supply table." But more anon.

---

*Meeting of the "American" Medical Association.*—Medical gentlemen representing several of the States met in this city a few days since, and nominally constituted a convention of the American Medical Association. Many of those in attendance supposed that the bold responsibility assumed by the Chairman of the Committee of Arrangements, was warranted and compulsory upon the organization. Others feared that if the meeting failed in point of numbers it would prove the funeral of the Association, and to prevent this they smothered their objections to the irregular usurpation of power which called the meeting, and met with the rest.

We shall have something to say about this meeting in a subsequent number of the Journal. *Now* we only say these things:

Meeting socially a large number of the delegates in attendance, we found but very few who did not regret the convening of the association at this time.

No important object whatsoever was accomplished by the gathering. There never has been a meeting of the association so generally ignored by the leading members of the profession throughout the country.

Its prominent act was a wholly gratuitous attack on the Sorgeon General ot the Army, an action which in its animus and results can only tend to bring the profession into contempt.

Politics and the canal convention were more talked and considered than medicine. Nevertheless, it still illustrated the strong good sense ,which is the glory of the profession by developing its opinion of the course taken by certain parties to this ill-timed call—even ignoring all precedent and custom in electing to offices and appointing to committees, only those who had nothing to do with the call. Truly, "the engineer was hoist with his own petard."

*Intra-Uterine Injections.*—Several recent cases of sudden death from intra-uterine injections have attracted the attention, not only of the profession at large, but even of the specialists. The same time it was supposed that the death resulted from the passage of the fluid or air into the abdominal cavity through the Fallopian tubes. More accurate observations have shown that it arose from introduction of air into the uterine sinuses.

The abortionists ascertained by fatal experience that puncturing the membranes was not only uncertain and tedious, but liable to the disagreeable accident of puncturing the uterus or even the iliac artery. The better posted class thereupon resorted to injections of carbonic acid, of air, of water or other fluid—the result was in either case now and then a death suddenly—in the first two cases from intentional introduction of air into the uterine cavity, and in the second place from defective apparatus or manipulation.

We have the details of a case occurring in a large interior village of an adjoining state, a few weeks since, where the abortionist went to the house (at the request of husband and wife conjointly) and in half an hour thereafter the patient breathed her last. *Ten minutes after the abortionist went into the room to manipulate, the patient was dead. Post mortem* proved the presence of air in the heart. It came out at the Coroner's inquest that the brutal quack (not a physician though practicing as an *ismatic*) introduced a catheter or uterine trochar into the uterus and just before withdrawing it *blew forcibly through it into the uterus.* The victim immediately screamed out, became livid in the face, gasped and was dead. This was the rascal's own confession to the husband on the spot. Details of the case will be given after the case has gone upon its trial in the criminal court.

---

*Books Received.*—Cazeaux's Midwifery; Bond's Den al Medicines; Bowman's Medical Chemistry; Medical Student's Vade Mecum; Headland's Action of Medicine; Packard's Minor Surgery; Hamilton on Fractures and Dislocations. Notices next month.

---

VACCINE MATTER.—Fresh and reliable Vaccine Matter may be obtained by enclosing One Dollar and addressing

CHICAGO MEDICAL JOURNAL,
P. O. Drawer 5787, Chicago, Ill.

